# Introduction of the Grayscale Median for Ultrasound Tissue Characterization of the Transplanted Kidney

**DOI:** 10.3390/diagnostics11030390

**Published:** 2021-02-25

**Authors:** Camilo G. Sotomayor, Stan Benjamens, Hildebrand Dijkstra, Derya Yakar, Cyril Moers, Stephan J. L. Bakker, Robert A. Pol

**Affiliations:** 1 Department of Internal Medicine, Division of Nephrology, University of Groningen, 9700 RB Groningen, The Netherlands; c.g.sotomayor.campos@umcg.nl (C.G.S.); s.j.l.bakker@umcg.nl (S.J.L.B.); 2 Department of Radiology, Clinical Hospital of the University of Chile, University of Chile, CP 8380453 Santiago, Chile; 3 Department of Surgery, Division of Transplant Surgery, University of Groningen, 9700 RB Groningen, The Netherlands; s.benjamens@umcg.nl (S.B.); c.moers@umcg.nl (C.M.); 4 Medical Imaging Center, Department of Nuclear Medicine and Molecular Imaging & Department of Radiology, University of Groningen, 9700 RB Groningen, The Netherlands; h.dijkstra01@umcg.nl (H.D.); d.yakar@umcg.nl (D.Y.)

**Keywords:** ultrasound, grayscale median, kidney transplant, kidney function, graft function

## Abstract

Ultrasound examination is advised for early post-kidney transplant assessment. Grayscale median (GSM) quantification is novel in the kidney transplant field, with no systematic assessment previously reported. In this prospective cohort study, we measured the post-operative GSM in a large cohort of adult kidney transplant recipients (KTR) who consecutively underwent Doppler ultrasound directly after transplantation (within 24 h), compared it with GSM in nontransplanted patients, and investigated its association with baseline and follow-up characteristics. B-mode images were used to calculate the GSM in KTR and compared with GSM data in nontransplanted patients, as simulated from summary statistics of the literature using a Mersenne twister algorithm. The association of GSM with baseline and 1-year follow-up characteristics were studied by means of linear regression analyses. In 282 KTR (54 ± 15 years old, 60% male), the median (IQR) GSM was 55 (45–69), ranging from 22 to 124 (coefficient of variation = 7.4%), without differences by type of donation (*p* = 0.28). GSM in KTR was significantly higher than in nontransplanted patients (*p* < 0.001), and associated with systolic blood pressure, history of cardiovascular disease, and donor age (std. β = 0.12, −0.20, and 0.13, respectively; *p* < 0.05 for all). Higher early post-kidney transplant GSM was not associated with 1-year post-kidney transplant function parameters (e.g., measured and estimated glomerular filtration rate). The data provided in this study could be used as first step for further research on the application of early postoperative ultrasound in KTR.

## 1. Introduction

Kidney transplantation is the optimal treatment strategy for patients with end-stage renal disease (ESRD), as it improves both patient survival and quality of life [1]. To ensure adequate kidney transplant function and transplant survival, comprehensive post-transplant follow-up is pivotal. Besides regular measurement of serum creatinine and glomerular filtration rate (GFR), ultrasound examination is advised as part of the post-transplant assessment [2,3].

Post-transplant ultrasound is primarily used for the exclusion of vascular (i.e., transplant renal artery stenosis and arteriovenous fistula) and urological (i.e., ureteral obstruction and urinoma) complications [4]. Besides this visual grayscale ultrasound examination, the most commonly used ultrasound technique after transplantation is measurement of the intrarenal resistance index (RI), by color and spectral Doppler [4,5]. Studies focusing on the use of RI measurements in this patient population reported strong associations with pre- and post-transplant patient characteristics [6,7]. In addition, associations with post-transplant outcomes, such as transplant and patient survival, were described [8,9].

A novel ultrasound technique for the field of kidney transplantation is ultrasound tissue characterization by means of grayscale median (GSM) quantification. This is a well-known technique within the field of vascular surgery, as it is commonly applied for the examination of carotid artery plaques [10,11,12]. A first overview of GSM reference values for native kidneys of healthy volunteers was provided by Engelhorn et al. [13], with GSM-values ranging approximately between 30 and 50. A relatively small study (*n* = 18), focusing on unilateral renal artery stenosis in native kidneys, reported an increase in GSM values compared to a situation without renal artery stenosis [14]. The application of ultrasound tissue characterization after kidney transplantation was only described in a single case report, showing a relatively high GSM at day one post-transplant, with an increase at seven days follow-up, in a case of biopsy-confirmed kidney transplant rejection [15].

To date, early postoperative ultrasound examinations are part of standard care after kidney transplantation [2,3]. As the application of the ultrasound tissue characterization technique is novel for this field, establishing reference values for the transplanted kidney is required for future investigations. Here, we present the results of a large cohort study on post-operative ultrasound tissue characterization by means of calculation of the GSM of the transplant kidney and investigate its association with kidney transplant outcomes at 1-year post-transplantation.

## 2. Materials and Methods

### 2.1. Study Design and Population

We performed a post-hoc analysis of a prospective cohort study performed in adult (>18 years old) kidney transplant recipients, who consecutively underwent Doppler ultrasound with RI measurement directly after transplantation. In this cohort study, patients were included at the University Medical Center Groningen (UMCG; Groningen, The Netherlands) between November 2015 and September 2017 (*n* = 364). Patients were excluded if flow measurements were insufficiently reported (*n* = 18), or if patients underwent a combined liver–kidney or kidney–pancreas transplantation (*n* = 7), leaving 339 patients eligible for further analyses. For the current post-hoc analyses, ultrasound tissue characterization by means of GSM measurement was performed using B-mode images obtained during post-operative (within 24 h post-transplantation) ultrasound examination of 282 kidney transplant recipients, of which data are presented here.

### 2.2. Data Collection

Electronic health records were screened to document patients’ baseline characteristics. Due to the descriptive character of this study, our institution’s Medical Ethics Committee granted dispensation for the Dutch law regarding patient-based medical research (WMO) obligation (Medical Ethical Committee UMCG-201800363, 3 September 2018). Patient data were processed and electronically stored according to the Declaration of Helsinki for medical research involving human subjects. The clinical and research activities were consistent with the Principles of the Declaration of Istanbul as outlined in the Declaration of Istanbul on Organ Trafficking and Transplant Tourism.

### 2.3. Ultrasound and Measurement of the Grayscale Median

**Figure 1:**
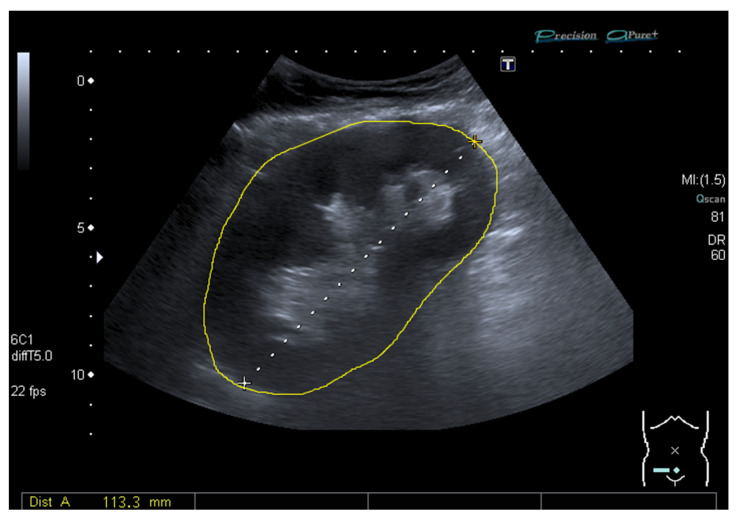
B-mode image from a common ultrasound of a transplanted kidney manually selected for calculation of the grayscale median (50).

**Figure 2:**
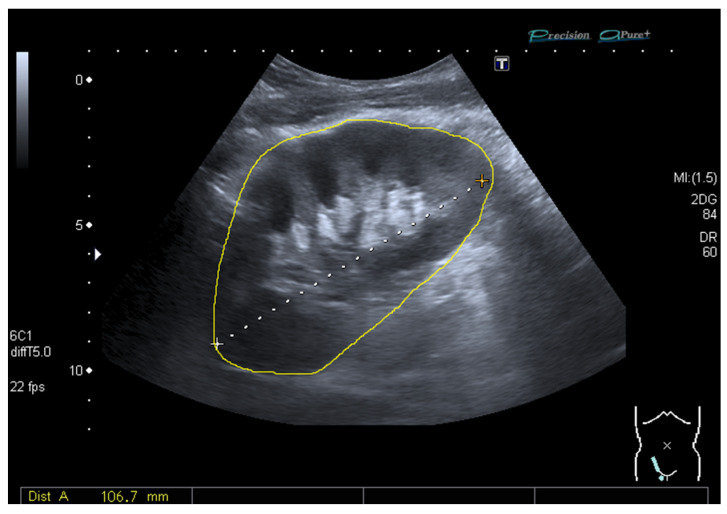
B-mode image from a common ultrasound of a transplanted kidney manually selected for calculation of the grayscale median (77).

Standard kidney ultrasound was performed within 24 h post-transplantation with a curved array transducer (multi-frequency 1–6 MHz) on a Toshiba Aplio MX (Tokyo, Japan) or Zonare ZS3 (Shenzhen, China) ultrasound system. Standard instrument preset determined the grayscale mapping used. Minimal setting adjustments were made (image depth 10–12 cm, total gain 65–85, dynamic range 60–65 dB, tissue harmonic image 5 MHz). The ultrasound examinations were performed by the on-call radiologists or radiology resident (supervised by an abdominal radiologist) and included examination of renal artery and vein anastomoses, kidney size, and RI (measured three times in the upper pole, interpolar, and lower pole in each patient). As previously described [7], the arterial RI was calculated as (peak systolic velocity − end diastolic velocity)/peak systolic velocity. In line with the methodology introduced by Engelhorn et al., US-based virtual histology analysis of carotid plaque was adapted for the kidney by means of pixel brightness analysis to calculate the GSM [13,15]. A freely available and extensively used image processing and analysis software (ImageJ; a public-domain Java-based image processing and analysis software developed by Wayne Rasband of the National Institute of Mental Health at NIH) was used for pixel brightness analysis of the recorded images (jpeg). The physician analyst (C.G.S.) manually selected a bright fascia to represent the 256 level of a 0–255 image range (Figure 1 and Figure 2). No changes from standard preset were followed in order to minimize setting variability, and the image analyses protocol did not include improving human visual perception of the image.

### 2.4. Statistical Analyses

Data were analyzed using IBM SPSS software version 26.0 (SPSS Inc., Chicago, IL, USA), R version 1.3.959 (R Foundation for Statistical Computing, Vienna, Austria) with standard packages, and GraphPad Prism 7.02 (GraphPad Software Inc., San Diego, CA, USA). Data were expressed as mean ± standard deviation (SD) for normally distributed variables, and as median (interquartile range; IQR) for skewed variables. Categorical data were expressed as *n* (percentage). In all analyses, a two-sided *p* < 0.05 was considered significant.

Differences in GSM among types of donors (donation after circulatory death (DCD), donation after brain death (DBD), and living donation) were tested with the Kruskal–Wallis test (used for comparing two or more independent samples with equal or different sample sizes) [16]. The GSM in nontransplanted patients was simulated with data from summary statistics reported by Engelhorn et al., using a Mersenne twister algorithm as proposed by Matsumoto et al. [13,17]. Linear regression analyses were performed to examine the association of baseline characteristics with GSM. Standardized β coefficients represent the difference (in SD) in GSM per 1 SD increment in continuous characteristics or for categorical characteristics in GSM compared with the implied reference group. Residuals were checked for normality and naturally log-transformed when appropriate. To study which baseline variables were independently associated with GSM and which were determinants, we performed forward selection of baseline characteristics according to preceding multivariable linear regression analyses (*p* for inclusion < 0.2), followed by stepwise backward multivariable linear regression analyses (*p* for exclusion < 0.05).

## 3. Results

### 3.1. Study Population

A total of 282 kidney transplant recipients were included. At baseline, mean age ± SD was 54 ± 15 years and 170 (60%) patients were male (Table 1). A pre-emptive transplantation was performed in 83 (28%) recipients. Patients who were dialysis-dependent had a median dialysis vintage pretransplant of 22 (IQR, 13–37) months. Fifty-nine (21%) patients had a history of smoking and 65 (23%) experienced a cardiovascular event prior to transplantation. Mean donor age was 54 ± 13 years and 152 donors (54%) were male. The distribution in terms of donor type was 166 (59%) living kidney donors, 66 (22%) donations after brain death (DBD), and 50 (18%) DCD. Further baseline characteristics of the study participants are shown in Table 1.

### 3.2. Grayscale Median in Kidney Transplant Recipients and Nontransplanted Patients

**Figure 3:**
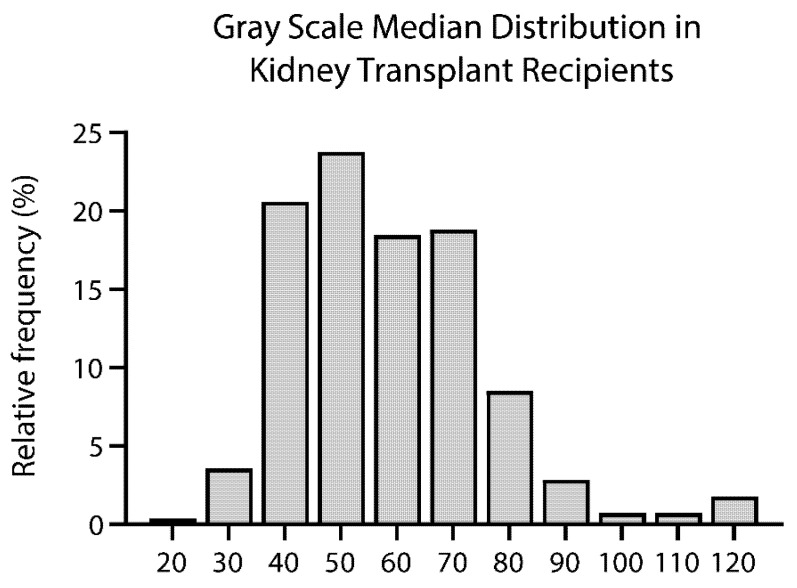
Histogram of grayscale median distribution in 282 kidney transplant recipients.

**Figure 4:**
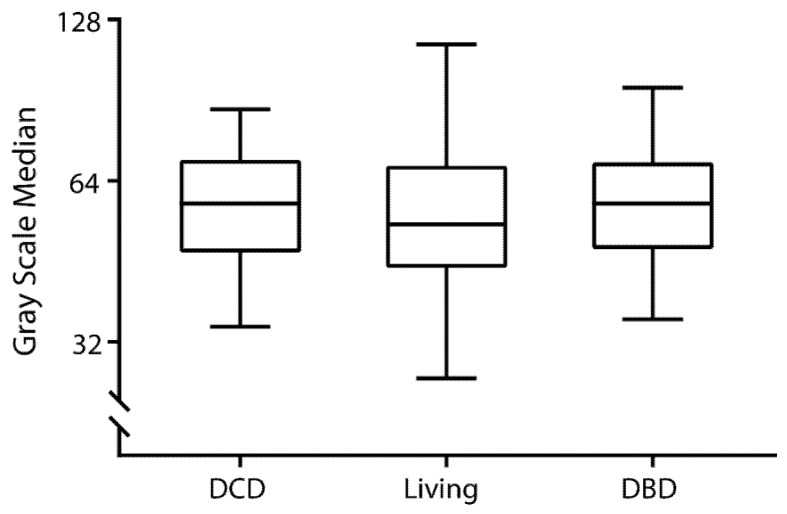
Grayscale median according to type of kidney donation, i.e., donation after cardiac death (DCD, *n* = 66), living donation (*n* = 166), and donation after brain death (DBD; *n* = 50). Significance of potential difference was tested using the Kruskal–Wallis test (*p* = 0.28).

**Figure 5:**
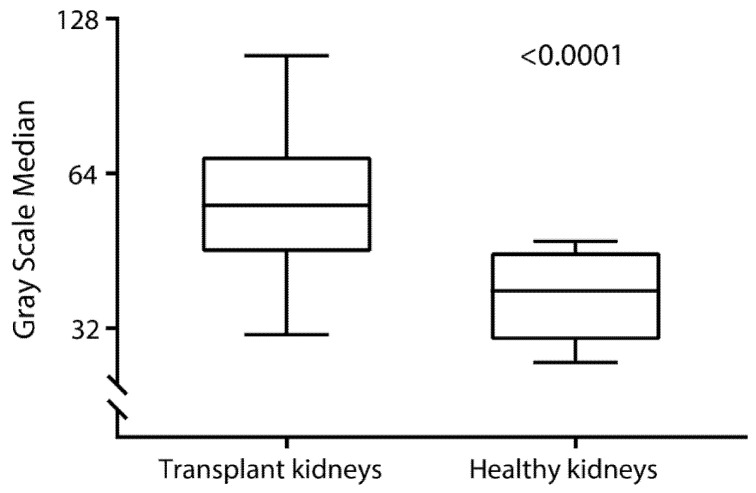
Grayscale median in kidney transplant recipients (mean 58 ± 18) and non-transplanted patients (mean 37 ± 6). Grayscale median was significantly different between kidney transplant recipients and healthy kidneys. Difference between groups was tested using the Mann-Whitney U test (*p* < 0.001).

The median GSM was 55 (IQR, 45–69), ranging from 22 to 124, resulting in a CV equal to 7.4%. Figure 3 shows GSM distribution in the study population of kidney transplant recipients. There was no statistically significant difference (*p* = 0.28) between the values obtained in transplanted kidneys from living (53 (44–68)), DCD (58 (47–71)), and DBD (58 (48–69)) donation (Figure 4). Figure 5 shows that GSM in kidney transplant recipients was significantly higher (*p* < 0.001) compared to the GSM in nontransplanted patients.

### 3.3. Cross-Sectional Analyses between Grayscale Median and Transplant Characteristics

In linear regression analysis (Table 2), we found an association of the GSM with systolic blood pressure (std. β = 0.12; *p* = 0.03), history of cardiovascular disease (std. β = −0.20; *p* = 0.003), and donor age (std. β = 0.13; *p* = 0.048). In stepwise, backwards, linear regression analysis the association of GSM with donor age was consistently found (std. β = 0.39; *p* = 0.02).

### 3.4. Association between Grayscale Median and Kidney Function Parameters at 1-Year Post-Transplant

In linear regression analyses between GSM and kidney function parameters (measured glomerular filtration rate, serum creatinine, creatinine clearance and estimated glomerular filtration rate) at 1-year post-transplant, we did not find any significant associations (Table 3).

## 4. Discussion

In the current study, we found that early post-operative GSM of kidney transplant recipients was qualitatively and quantitatively higher than in kidneys of nontransplanted patients previously reported in the literature [13]. Interestingly, we also found that, in kidney transplant recipients, GSM values were positively associated with donor age and kidney transplant recipient blood pressure and inversely with kidney transplant recipient history of cardiovascular disease.

With early post-operative ultrasound examinations as part of standard care after kidney transplantation, visual ultrasound interpretation by means of B-mode inspection is part of daily practice. This inspection of B-mode ultrasound images focuses on signs of complications, such as enlargement of the transplanted kidney, fluid collections, and the iliac and transplant vasculature [18]. It may also allow detection of morphological changes, alterations in the echogenicity of the graft and distinctive structures, such as medullary pyramids, and overall allograft swelling [19]. As is generally accepted for all human interpretations of radiological images, the interpretation of B-mode images is prone to interobserver variability. To combat interobserver variability due to evaluation of arbitrary observations, the GSM ultrasound technique was originally introduced as a quantitative measure for vascular ultrasound interpretation [20]. For kidney ultrasound, this technique was adapted by Engelhorn et al., with only limited data about transplanted kidney GSM values available, until now [13]. To our knowledge, this is the first cohort study reporting on ultrasound B-mode GSM measurement of transplant kidneys systematically performed early post-operation (within 24 h post-transplantation). The observed levels of GSM in this study were consistent with a previous case report of kidney transplant rejection, in which GSM was high, and with an observation of 31 symptomatic kidney transplant recipients who had abnormally hyperechoic transplanted kidneys, with GSM in the range of 51–83 [13,15]. Our systematic measurement of early post-operative kidney graft GSM provides a reference and solid database for future comparison of ultrasound B mode GSM examination of transplanted kidneys of potential clinical use, raising suspicions of abnormality in case of extremely high or low echogenicity. 

We observed that GSM values were higher compared to previous reports in nontransplanted patients [13], which may be indicative of typical underlying pathological processes of transplanted kidneys, such as ischemia–reperfusion, acute tubular necrosis, glomerulonephritis, and acute organ rejection. It should be realized, however, that substantial intraindividual variability was found among kidney transplant recipients in the current study. Although there was a trend toward a positive association between GSM and adverse early post-transplant outcomes (e.g., delayed graft function, comprehensive complication index, and length of hospital stay), we did not find signs of a significant association between GSM and 1-year post-transplant kidney function parameters. The observed high intraindividual variability of GSM among kidney transplant recipients was in line with previous experiences using ultrasound-derived parameters measured within a short time-frame after the kidney transplant surgical procedure, wherein observations of large variability largely dominated and may have impacted the prognostic capacity of ultrasound-derived quantitative features. We hypothesize that a GSM assessed early after kidney transplantation may not yet reflect the installation of foregoing pathological processes associated with chronic organ damage and longitudinal graft function decline, but rather uncompromising factors associated with the kidney transplant procedure itself, such as edema and mild hydronephrosis [21,22]. This prevailing post-operative organ response may obscure visually subtler yet pathophysiologically major detrimental processes at this stage, making an early post-operative GSM measurement less suitable to evaluate outcome prognoses than GSM measurements performed at a later stage during follow-up of seemingly stable outpatient kidney transplant recipients. In the latter clinical setting, a higher GSM may more precisely serve to quantify corticomedullary differentiation, increased parenchymal echoes, or increased cortical reflectivity [22]. Particularly in patients at increased risk of graft loss, as determined by means of a high Maryland Aggregate Pathology Index [23], for instance, monitoring kidney graft GSM could offer a cost-efficient follow-up imaging approach to aid in decision-making for performing further examinations (e.g., by MRI or histology). Further studies are warranted to evaluate this hypothesis. 

Strengths of this study include the standard protocol for routine kidney ultrasound within 24 h post-transplantation, the inclusion of kidney transplant recipients with living (un)related donors, DBD and DCD kidneys, and the relatively large sample size, enabling multivariable linear regression analyses. The main limitation of this study was the short period of follow-up, restricting the analysis to 1-year post-transplant. Because routine reperfusion biopsies are not performed anymore, histopathological correlation with early post-transplant ultrasound findings is not possible. The GSM ultrasound technique was applied in a retrospective manner, using previously saved and stored kidney ultrasound images. The kidney ultrasound images were made by the on-call radiologists (in-training) or radiology resident (supervised by an abdominal radiologist), resulting in a variety of medical doctors performing the examinations. As for all observational studies, multivariable analyses cannot completely rule out potential residual confounding. To note, the stratification used for this study, as tertiles based on group size, is highly population dependent. This stratification should only be applied in transplant recipient populations with similar demographics; specific cut-off values should be determined otherwise.

In conclusion, in early post-operative kidney transplant recipients, ultrasound tissue characterization results in a higher GSM compared to nontransplanted patients. The data provided in this study can be used as a first step for further research on the application of ultrasound tissue characterization after kidney transplantation.

## Figures and Tables

**Table 1:** Baseline characteristics of the overall population of kidney transplant recipients, and by tertiles of grayscale median (GSM).

Baseline Characteristics	Overall	Tertiles of GSM			*p*
	(*n* = 282)	Tertile 1 (*n* = 94)	Tertile 2 (*n* = 94)	Tertile 3 (*n* = 94)	
GSM	55 (45–69)	42 (38–45)	56 (51–60)	73 (69–81)	-
**Recipients characteristics**					
Age, years, mean ± SD	54 ± 15	54 ± 15	55 ± 14	54 ± 16	0.84
Sex, male, *n* (%)	170 (60)	49 (52)	60 (64)	61 (65)	0.14
BMI, kg/m^2^, mean ± SD	26.2 ± 4.3	26.5 ± 4.9	25.9 ± 4.0	26.0 ± 4.1	0.60
Systolic blood pressure, mmHg, mean ± SD	143 ± 21	138 ± 20	142 ± 19	148 ± 23	0.005
Diastolic blood pressure, mmHg, mean ± SD	80 ± 13	79 ± 12	80 ± 13	82 ± 13	0.23
Use of antihypertensives, *n* (%)	211 (75)	70 (75)	75 (80)	66 (70)	0.32
Pre-emptive transplantation, *n* (%)	83 (28)	33 (35)	22 (23)	28 (30)	0.21
Dialysis vintage, months, median (IQR)	22 (13–37)	23 (16–37)	20 (12–34)	23 (13–41)	0.76
Smoking history, *n* (%)	59 (21)	21 (22)	15 (16)	23 (25)	0.33
History of DM, *n* (%)	57 (20)	17 (18)	17 (18)	23 (25)	0.28
History of CVD, *n* (%)	65 (23)	30 (32)	19 (20)	16 (17)	0.02
**Donors characteristics**					
Age, years, mean ± SD	54 ± 13	52 ± 16	56 ± 11	53 ± 13	0.15
Sex, male, *n* (%)	152 (54)	52 (55)	52 (55)	48 (51)	0.56
BMI, kg/m^2^, mean ± SD	26.0 ± 3.8	25.7 ± 4.4	26.2 ± 3.4	26.0 ± 3.4	0.70
Systolic blood pressure, mmHg, mean ± SD	127 ± 18	127 ± 17	126 ± 19	128 ± 19	0.71
Diastolic blood pressure, mmHg, mean ± SD	73 ± 11	73 ± 11	71 ± 11	74 ± 11	0.05
Use of antihypertensives, *n* (%)	26 (9)	10 (11)	10 (11)	6 (6)	0.50
**Type of donation**					
Living donor, *n* (%)	166 (59)	62 (66)	51 (54)	53 (56)	0.22
DCD, *n* (%)	66 (22)	19 (20)	23 (25)	24 (26)	0.66
DBD, *n* (%)	50 (18)	13 (14)	20 (21)	17 (18)	0.41
**Transplant characteristics**					
Side donor kidney, right, *n* (%)	85 (30)	32 (34)	32 (34)	21 (22)	0.10
Cold ischemia time, minutes, median (IQR)	30 (16–41)	29 (17–40)	33 (16–43)	31 (15–44)	0.64
Cold ischemia time, >12 h, *n* (%)	56 (20)	16 (17)	21 (22)	19 (20)	0.66
**Ultrasound**					
Time between PO and US, minutes, median (IQR)	53 (29–86)	62 (35–114)	50 (28–85)	49 (29–76)	0.08
Position of the kidney, right iliac fossa, *n* (%)	228 (81)	77 (82)	74 (79)	77 (82)	0.76
Central resistive index, mean ± SD	0.68 ± 0.09	0.68 ± 0.08	0.69 ± 0.08	0.67 ± 0.10	0.60
Peripheral resistive index, mean ± SD	0.64 ± 0.08	0.64 ± 0.08	0.65 ± 0.08	0.64 ± 0.09	0.70
**Post-operative outcomes**					
DGF, *n* (%)	49 (17)	12 (13)	16 (17)	21 (22)	0.22
DGF, days, median (IQR)	9 (6–12)	8 (6–9)	6 (4–12)	10 (7–13)	0.13
CCI, median (IQR)	9 (0–23)	9 (0–21)	9 (0–23)	9 (0–23)	0.45
Length of hospital stay, days, median (IQR)	8 (7–11)	8 (7–10)	8 (7–11)	8 (6–13)	0.77

Differences were tested by analysis of variance or Kruskal–Wallis test for continuous variables and by chi-squared test for categorical variables. BMI, body mass index; DM, diabetes mellitus; CVD, cardiovascular disease; CCI, comprehensive complication index; DBD, donation after brain death; DCD, donation after circulatory death; DGF: delayed graft function.

**Table 2:** Association between grayscale median and baseline characteristics in 282 kidney transplant recipients.

Characteristics	Unadjusted Linear Regression		Stepwise Backwards Linear Regression	
	Std. β	*p*	Std. β	*p*
**Recipients characteristics**				
Age, years	0.04	0.56		
Sex, male, *n*	−0.08	0.26		
BMI, kg/m^2^	−0.06	0.34		
Systolic blood pressure, mmHg	0.15	0.03	-	
Diastolic blood pressure, mmHg	0.02	0.78		
Use of antihypertensives, *n*	−0.04	0.55		
Pre-emptive transplantation, *n*	−0.001	0.99		
Dialysis vintage, months	0.03	0.72		
Smoking history, *n*	0.07	0.29		
History of DM, *n*	0.06	0.40		
History of CVD, *n*	−0.20	0.003		
**Donors characteristics**				
Age, years	0.13	0.05	0.39	0.02
Sex, male, *n*	0.03	0.39		
BMI, kg/m^2^	0.05	0.48		
Systolic blood pressure, mmHg	0.06	0.37		
Diastolic blood pressure, mmHg	0.02	0.76		
Use of antihypertensives, *n*	0.01	0.90		
**Type of donation**				
Living donor, *n*	−0.01	0.87		
DCD, *n*	0.01	0.92		
DBD, *n*	0.01	0.92		
**Transplant characteristics**				
Side donor kidney, right, *n*	−0.04	0.58		
Cold ischemia time, minutes	0.03	0.65		
Cold ischemia time, >12 h, *n*	0.04	0.56		
**Ultrasound**				
Time between PO and US, minutes	−0.11	0.10	-	
Position of the kidney, right iliac fossa, *n*	−0.03	0.67		
Central resistive index	0.01	0.84		
Peripheral resistive inde	−0.06	0.41		
**Follow-up**				
DGF, *n*	0.11	0.11	-	
DGF, days	0.23	0.17	-	
CCI	0.05	0.53		
Length of hospital stay, days	0.09	0.16	-	

Unadjusted linear regression analyses of the association between grayscale median and kidney function parameters. Coefficients represent the difference (in SD) in grayscale median per 1 SD increment in kidney function parameters. BMI, body mass index; CCI, comprehensive complication index; DM, diabetes mellitus; CVD, cardiovascular disease; DBD, donation after brain death; DCD, donation after circulatory death; DGF, delayed graft function.

**Table 3:** Association between GSM and kidney function parameters at 1-year post-transplantation.

Kidney Function Parameters	Linear Regression Analyses	
	Std. β	*p*
Glomerular filtration rate	−0.07	0.35
Serum creatinine	0.003	0.97
Creatinine clearance	0.02	0.82
Estimated glomerular filtration rate	−0.02	0.76

Unadjusted linear regression analyses of the association between grayscale median and kidney function parameters. Coefficients represent the difference (in SD) in grayscale median per 1 SD increment in kidney function parameters.

## Data Availability

Data available on request due to privacy restrictions.
